# Moving Healthcare Quality Forward With Nursing-Sensitive Value-Based Purchasing

**DOI:** 10.1111/j.1547-5069.2012.01469.x

**Published:** 2012-12

**Authors:** Kevin T Kavanagh, Jeannie P Cimiotti, Said Abusalem, Mary-Beth Coty

**Affiliations:** 1 Board Chairman, Health Watch USASomerset, KY; 2 Alpha Zeta, Alpha Tau, and Phi Gamma, Associate Professor, Rutgers University College of NursingNewark, NJ; 3 Assistant Professor, University of Louisville, and Researcher, Health Watch USALouisville, KY; 4 Assistant Professor, University of LouisvilleLouisville, KY

**Keywords:** Adverse events, nursing-sensitive, quality measures, value-based purchasing

## Abstract

**Purpose:** To underscore the need for health system reform and emphasize nursing measures as a key component in our healthcare reimbursement system.

**Design and Methods:** Nursing-sensitive value-based purchasing (NSVBP) has been proposed as an initiative that would help to promote optimal staffing and practice environment through financial rewards and transparency of structure, process, and patient outcome measures. This article reviews the medical, governmental, institutional, and lay literature regarding the necessity for, method of implementation of, and potential impact of NSVBP.

**Findings:** Research has shown that adverse events and mortality are highly dependent on nurse staffing levels and skill mix. The National Database of Nursing Quality Indicators (NDNQI), along with other well-developed indicators, can be used as nursing-sensitive measurements for value-based purchasing initiatives. Nursing-sensitive measures are an important component of value-based purchasing.

**Conclusions:** Value-based purchasing is in its infancy. Devising an effective system that recognizes and incorporates nursing measures will facilitate the success of this initiative. NSVBP needs to be designed and incentivized to decrease adverse events, hospital stays, and readmission rates, thereby decreasing societal healthcare costs.

**Clinical Relevance:** NSVBP has the potential for improving the quality of nursing care by financially motivating hospitals to have an optimal nurse practice environment capable of producing optimal patient outcomes by aligning cost effectiveness for hospitals to that of the patient and society.

Despite spending more on health care than any other nation in the world, the United States continues to perform poorly in healthcare quality when compared with other industrialized nations. To address this disparity, the Patient Protection and Affordable Care Act (PPACA) became law and directed the Centers for Medicare & Medicaid Services (CMS) to initiate a value-based purchasing system to financially incentivize healthcare quality and lower societal healthcare costs. The PPACA provides only an outline for change. A proactive stance in health policy formulation is needed that is based on the best information available. To achieve this goal, this policy report analyzes and expands the concept of nursing-sensitive value-based purchasing (NSVBP) as a mechanism to financially incentivize organizations for adequate nurse staffing that leads to high-quality patient care. At the heart of NSVBP is the concept that one cannot have a quality institution without providing highly trained and skilled nursing care. Yet nursing is at risk for inadequate support, both in staffing numbers and skill mix, particularly in today's cost-driven and financially stressed healthcare delivery systems.

## The Need for Incentives to Promote Healthcare System Reform

The current healthcare system in the United States continues to have sharply rising costs and lower quality compared with other industrialized countries on measures of preventable mortality, number of uninsured, and care system efficiency scores (Davis, Schoen, & Stremikis, 2010). Healthcare spending is predicted to comprise 20% of the U.S. gross national product by 2019 (Alonso-Zaldivar, 2010). The United States also pays far more per capita than any other nation, spending 81% more for health care than the average Organisation for Economic Co-operative Development (OECD) nation (OECD, 2011). The largest and growing component of the U.S. healthcare system is hospital care. Inpatient and outpatient services now represent 32% and 18% of the U.S. healthcare budget, respectively (Milliman, 2012). Associated with healthcare reform, integration and mergers are taking place (Moody's Investors Service, 2012) with insurers, for-profit companies, and private equity firms entering into the healthcare delivery market (Caramenico, 2012). This integration, despite hopes for widespread savings through the efficiency of size, has been associated with rising healthcare costs (Berenson, Ginsburg, Christian, & Yee, 2012; Office of Attorney General Martha Coakley, 2010) and a system-wide decrease in patient satisfaction. A recent poll of U.S. residents with illness found that 73% felt that the cost of health care was a very serious problem and 45% felt that quality was a very serious problem (National Public Radio, Robert Wood Johnson Foundation, Harvard School of Public Health [NPR/RWF/Harvard], 2012). This perception is supported by the finding that the United States has a below-average life expectancy rate and above-average infant mortality rate compared with the average OECD nation (OECD, 2011). Quality checks and balances are also at risk of disappearing. New CMS regulations (CMS, 2012) allow many multihospital systems to dissolve their local community hospital board, and the increasing employment of physicians by hospitals (Kocher & Sahni, 2011) has resulted in the loss of the independent medical staff at many institutions.

Two recent studies have found that approximately one in seven hospitalized patients is harmed by an adverse event (Landrigan et al., 2010; Office of Inspector General [OIG], 2010), and 44% to 66% of these events were judged as preventable. The hospital's administration may not be aware these events have even occurred, as evidenced by the OIG report that only 14% of events that cause harm are captured by hospital tracking systems (OIG, 2012). Downey, Hernandez-Boussard, Banka, and Morton (2012) examined the Agency for Healthcare Research and Quality (AHRQ) patient safety indicators between the years 1988 and 2007 and observed little overall change during the past decade—an ineffective response to the publication of the 1999 report from the Institute of Medicine (IOM), which estimated that almost 100,000 patients die each year from medical errors (IOM, 2000).

The magnitude, severity, and cost of healthcare errors continue to be unacceptability high. For example, healthcare-associated infections (HAIs) afflict 1.7 million hospitalized patients each year (Klevens et al., 2007), approximately 1 in 20 patients, at a cost to the U.S. healthcare system of $35.7 to $45.0 billion each year (Scott, 2009), causing nearly 100,000 deaths and untold disability (Klevens et al., 2007). The recent NPR/RWF/Harvard (2012) survey found that 8% of hospitalized patients report getting an HAI. The healthcare system continues to face many challenges in soaring costs and low quality of care, as evidenced by the slow progress in reducing adverse events. In response, CMS, under the PPACA, along with some private insurance companies, are well underway in implementing financial incentives that reward good quality and penalize bad.

## Nursing as an Indispensable Key Component of a Quality Healthcare System

As stated by the American Nurses Association (ANA), nurses are “the largest direct provider of care impacting patient outcome” (Montalvo, 2008, p. 3). A decade ago, Aiken, Clarke, Sloane, Sochalski, and Silber (2002) documented this critical role. They observed that for each additional patient a nurse cared for there was a 7% increase in patient mortality, and increasing patient loads, from one nurse for every four patients to one nurse for every eight patients, increased the risk for death by 31%.

Similar findings were reported the same year, with the observation that increased registered nurse staffing resulted in lower rates of urinary tract infections, upper gastrointestinal bleeding, pneumonia, shock, cardiac arrest, and failure to rescue (Needleman, Buerhaus, Mattke, Stewart, & Zelevinsky, 2002). Kovner, Jones, Zhan, Gergen, and Basu (2002) reported lower rates of postoperative pneumonia with an increase in registered nurse staffing. Seago (2001) reported that there is a strong association between a reduction in nursing staff and “increased length of stay, nosocomial infection (urinary tract infection, postoperative infection, and pneumonia), and pressure ulcers” (p. 426). Dunton, Gajewski, Taunton, and Moore (2004) and Patrician et al. (2011) substantiated the importance of registered nurse staffing in the prevention of falls.

The importance of nursing in the prevention of pressure ulcers and falls cannot be overstated. Prevention of pressure ulcers is a complex task, involving a daily risk assessment, proper nutrition, pressure redistribution, and moisture management, along with being sure the patient is not overmedicated and sedated. Similarly, the prevention of falls also is dependent on patient risk assessment, nutrition, supervised ambulation, assistive devices, and not overmedicating or sedating the patient.

In 2002, The Joint Commission identified nurse staffing levels as a factor in nearly 24% of sentinel events. Only 66% of nurses reported their units were adequately staffed with registered nurses and that “care is literally being left undone” (The Joint Commission, 2002, p. 11). Units with the most missed nursing care have reported “great difficulty with staffing adequacy on all shifts” (Kalisch, Gosselin, & Choi, 2012, p. 3). Today, undelivered care is still associated with low levels of staffing. However, to achieve the highest level of care, adequate staffing levels, and teamwork with trust, accountability, and impeccable leadership must also be present (Kalisch, Gosselin, et al., 2012). There is a significant negative correlation between teamwork and the number of nursing assistants, and the facility's daily census (Kalisch, Russell, & Lee, 2012). Teamwork and the work environment are important factors in the prevention of death among hospitalized patients (Aiken et al., 2011). Cimiotti, Aiken, Sloane, and Wu (2012) have recently reported the association between nurse burnout and HAIs, finding that for each additional patient above 5.7 that a nurse was responsible for, there was an increase in the rate of catheter-associated urinary tract infections by 1 per every 1,000 patients and surgical site infections by 5 per every 1,000 patients.

Little appears to have changed as evidenced by the NPR/RWF/Harvard (2012) survey, which found that one third of patients hospitalized in the past year reported that nurses did not respond quickly to requests or were not available when needed.

## Nursing Is at Risk for Being Cut in Financially Stressed, Cost-Driven Healthcare Delivery Systems

As described in “The Cost Conundrum Redux” by Atul Gawande (2009) and as evidenced by the vast differences in healthcare utilization between Medicare referral regions, which cannot be explained away by population health disparities (Fisher et al, 2003; Mittler, Landon, Fisher Cleary, & Zaslavsky, 2010; Song et al., 2010), a significant number of institutions appear to be cost driven. The downturn in the U.S. economy along with the provision of care for the uninsured and underinsured are straining the healthcare system. Facility administrators may decide not to hire nurses to meet short-term operating budgetary goals. Staffing expenses range from 50% to 70% of a facility's operating budget (Barns, 2010; Jones, 2009; Schuhmann, 2008), and nursing salaries comprise more than half of the labor costs (Barns, 2010). In some facilities, nursing salaries account for almost 50% of the total operating budget of a hospital (Manojlovich, 2009). As early as 1986, hospital administrators believed that the most effective way to decrease a hospital's operating budget was by cutting nursing staff (Kirkpatrick, 1986). This view is still held today, with some hospitals resorting to layoffs, which in turn may increase nurse workload (Jones, 2009; Manojlovich, 2009).

When staff is cut to meet short-term budgetary goals, one would expect an increase in adverse events, which would create an increase cost to the facility. However, an increase in costs to society and the payer may be cost effective for the facility. Hospitals, unlike society, do not necessarily procure a significant financial benefit by decreasing adverse events and mortality by increasing their nursing staff (Shamliyan, Kane, Mueller, Duval, & Wilt, 2009). Rothschild, Bates, Franz, Soukup, and Kaushal (2009) calculated the annual reduction in hospital resources utilized (including both actual variable and actual fixed hospital costs) of nurse-intercepted and -prevented events in coronary care units to be between $2.2 and $13.2 million, compared with the nurse staffing cost of $1.36 million. It should be noted that a facility can save on the variable costs by decreasing adverse events, but not on fixed costs, which are unaffected by production. Rothschild et al. (2009) also commented, “ … with current reimbursement models, hospitals that expend more resources for better nurse staffing may not directly benefit financially” (p. 471.e6). Rothberg, Abraham, Lindenauer, and Rose (2005) have observed that increased staffing places a considerable financial burden on hospitals and the cost-effectiveness for the facility decreases as patient-to-nurse ratios decrease from 8:1 to 4:1. However, the average cost per life saved did not exceed $136,000, a relative bargain for society.

Similarly, avoidance of a readmission may save the patient and third-party payer money, but it may also cost the hospital income under the current reimbursement systems from the loss of future business. Increasing non-overtime staffing of registered nurses by just 45 min per patient day in 16 nursing units was observed to decrease hospital readmission and provided a yearly net healthcare savings (to the payer) of $11.64 million but had a negative impact on the facility (Weiss, Yakusheva, & Bobay, 2011).

Dall, Chen, Seifert, Maddox, and Hogan (2009) performed a systemwide analysis of financial data on the effect of nursing staffing on hospital and third-party payer healthcare costs. They concluded that if all U.S. hospitals staffed to at least the 75th percentile, there would be a 3.6 million national annual decrease in hospital days, a saving of 5,900 lives, and a savings to the patient in future work productivity of $1.3 billion. The medical (before increased labor costs) savings totaled $6.1 billion but cost the hospital an estimated $11 billion in labor costs. Needleman, Buerhaus, Stewart, Zelevinsky, and Mattke (2006) came to similar conclusions and found that an increase in registered nurse staffing, resulting in an increase in total nurse staffing hours, resulted in a decrease in adverse events but a net increase in hospital costs of 1.5%. They concluded that the cost effectiveness depends on the value that society places on avoidance of disability and loss of life. As stated by Dall et al. (2009), “More closely linking reimbursement to patient outcomes could help facilities capture more of the benefits from improved staffing … and provide the means to improve quality of care” (p. 104). This is the premise behind NSVBP.

## Types of Value-Based Purchasing

There are two types of value-based purchasing incentives: transparency and financial. Transparency of measures is important to allow consumers and referrers to make choices between different hospitals based on quality and performance. At least 27 states and the District of Columbia publicly report HAIs (Frieden, 2010). Data on hospital-acquired conditions (HACs), process measures, and patient satisfaction surveys are available to the public through Hospital Compare (CMS, n.d.). Public reporting of quality measures could become a factor in a facility's negotiation of insurance contracts with employer purchasing alliances and third-party payers. In addition, it could affect physician referrals and advice to patients regarding a facility. Transparency can have a profound indirect financial impact on a facility's revenues.

Financial incentives are of two categories: the first penalizes payment for the care given to an individual patient who acquires an HAC. The second involves penalizing or rewarding the entire fee schedule for all services and patients that are treated at a facility. The type of incentive that is chosen depends on whether a facility's reimbursement is a payment for individual (line item) services or a bundled payment.

Private insurance companies often pay for each individual service a patient receives. Under this system, patients who develop HACs are profitable to the hospital as opposed to an increase in mortality rates, which will cause a loss of income. Effective financial incentives would include both nonpayments for care related to HACs and penalization or reward of the facility's fee schedule.

The current Medicare Diagnostic Related Group system is a bundled payment based on the patient's major diagnosis at admission. Other bundled payment systems involving one payment per event or a modified capitation system (e.g., one payment per patient per year) are being spurred by the PPACA through the Accountable Care Organization initiative. In these types of bundled payment systems, HACs can result in a huge financial drain through expended resources, but an increase in mortality may result in a financial gain, since resources are not spent but the payment may remain the same. Penalties based on individual services provided to the patients would not be expected to be effective. For example, CMS's policy of nonpayment for care related to HACs has only recouped $18.8 million nationwide during the first year of the program, ending in September 2009 (CMS, 2010), a small pittance when compared with the costs presented in **Table 1**. The basis of this initiative was to prevent the hospital from receiving the maximum payment possible, but all too often another secondary diagnosis can be used to achieve this. The Commonwealth Fund has reported that this nonpayment initiative has not produced significant policy changes in safety net hospitals, with the majority of financial officers who developed an impact analysis stating the financial impact would be “minimal” or “inconsequential” (McHugh, Van Dyke, Osei-Anto, & Haque, 2011).

**Table 1:** Examples of Nursing-Sensitive Outcome Measures and the Estimated Annual Cost for the Adverse Events in Hospitals That These Measures Are Designed to Decrease

Outcome measures	NQF-endorsed measure reference number^*^	NDNQI indicator	CMS VBP	Annual cost of adverse events in hospitals
Infections				
SSIs	130 and 753		x	$3.45–10.07 billion (U.S. cost; Scott, 2009)
VAP	140	x		$1.03–1.50 billion (U.S. cost; Scott, 2009)
CLABSIs	139	x	x	$0.67–2.68 billion (U.S. cost; Scott, 2009)
CAUTIs	138	x	x	$0.39–0.45 billion (U.S. cost; Scott, 2009)
Decubitus ulcers, Stage III and V	337	x	x	$11.11 billion (Medicare cost; CMS, 2008)
Falls and falls with injury	141 and 202	x	x	$6.56 billion (Medicare cost, including burns; CMS, 2008)
Failure to rescue	352 and 353			
Patient mortality	231, 343, 347, 358, 530, 703, 730, and 733		x	
Length of stay in ICU	702			
Readmissions	1768, 1551, 330, 335, 505, 506, and 695		x	$15 billion (Medicare cost, Hackbarth, 2009)

*Note.* NQF = National Quality Forum; NDNQI = National Database of Nursing Quality Indicators; CMS =Centers for Medicare and Medicaid Services; VBP = value-based purchasing; SSI = surgical site infection; VAP = ventilator-associated pneumonia; CLABSI = central line-associated bloodstream infection; CAUTI = catheter-associated urinary tract infection; ICU = intensive care unit. ^*^Additional metrics than those listed have been defined by NQF.

High rates of patient readmissions may also generate income for an institution, but have disastrous results for the patient. It has been reported that one in five Medicare discharges are readmitted within 30 days (Jencks, Williams, & Coleman, 2009). The Medicare Payment Advisory Committee estimates that readmissions cost Medicare $15 billion annually, of which $12 billion is from potentially preventable readmissions (Hackbarth, 2009). CMS is set to reduce total Medicare payments to hospitals with high readmission rates by 1% in 2013, 2% in 2014, and 3% in 2015 (CMS, 2011a).

## Measures That Promote Quality Nursing Care: Nursing-Sensitive Value-Based Purchasing

Theoretically, NSVBP is based on Donabedian's (1966) structure-process-outcomes model, where structural characteristics are associated with the process of care and patient outcomes. The National Database of Nursing Quality Indicators (NDNQI) has been developed by the ANA for collection and evaluation of nursing-sensitive data used in the evaluation of nursing performance in relation to patient outcomes (NDNQI, 2011). In addition to the ANA, the National Quality Forum (NQF, 2011), AHRQ (2007), and CMS have endorsed a series of measures that are being considered in value-based purchasing initiatives.

### Structure Measures

As defined by the ANA (2011), “The structure of nursing care is indicated by the supply of nursing staff, the skill level of the nursing staff, and the education/certification of nursing staff” (Defined section, ¶ 1). Expenses to the hospital caused by nursing turnover are significant and accounted for greater than 5% of the operating budget in one academic medical center in the southwest (Waldman, Kelly, Arora, & Smith, 2004). Nurse retention rates or voluntary turnover rates are a nursing-sensitive indicator that would be of great benefit and addition to the current value-based purchasing initiatives.

Nurse staffing measures such as full-time equivalents, nursing hours per patient per day, or nurse-to-patient ratios are often analytically adjusted for patient acuity. There is evidence to suggest that the minimum staffing requirement in California has been effective in preventing adverse events and improving positive patient outcomes (Aiken et al., 2010; Donaldson & Shapiro, 2010). However, with fixed ratios, nurses may be expected to perform non-nursing tasks and the functions of ancillary employees (Manojlovich, 2009; Stanton & Rutherford, 2004).

A significant reduction in morbidity was reported with increases in registered nursing levels, but these findings did not hold true for increasing staffing levels of licensed practical nurses or nurses’ aides (Needleman et al., 2002). Thungjaroenkul, Cummings, and Embleton (2007) found that an increase in registered nurse staffing levels was associated with a significant reduction in length of stay and patient costs. Cost savings to the patient are inevitable with shortened hospitalizations and decreased complications. Recent findings suggest that reduced registered nurse staffing was associated with an increase in overall patient mortality (Needleman et al., 2011). Patrician et al. (2011) found that an increase in registered nurse skill mix, total nursing care hours, and nursing experience was associated with a lower incidence of adverse events.

**Figure 1:**
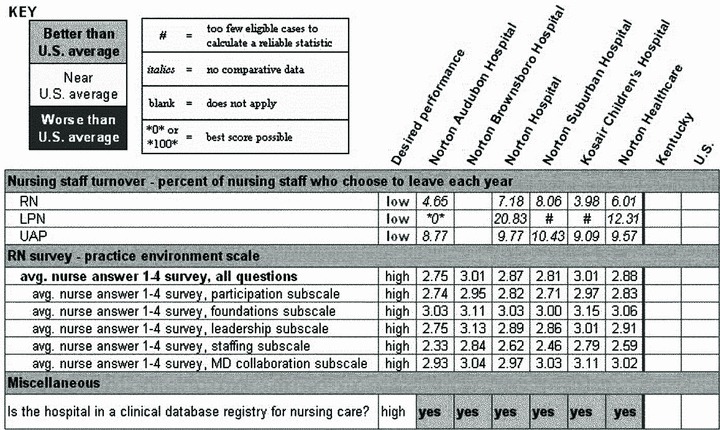
Public reporting of structure measures. Used with permission from Norton Healthcare.

Examples of structure measures, which are contained in the NDNQI, along with their NQF identification numbers, are shown in **Table 2**. Structure measures are publicly reported by the State of Illinois, including nursing and registered nurse hours per patient day, turnover rates, and hours worked by contractual registered nurses (Illinois Department of Public Health, n.d.), and the State of New Jersey reports skill mix and patient-to-nurse ratios (New Jersey Department of Health and Senior Services, n.d.). In addition, some hospitals voluntarily report these measures on their respective websites (Figure 1).

**Table 2:** Examples of Nursing Sensitive Structure Measures

Measure	NQF measure reference number	NDNQI-endorsed indicator
Voluntary turnover rate (nurse retention)	207	x
RN, LPN, and UNA skill mix	204	x
Practice Environment Scale	206	x
Nursing hours supplied by temporary staff		x
Hours of nursing care per patient day	205	x

*Note.* NQF = National Quality Forum; NDNQI = National Database of Nursing Quality Indicators; RN = registered nurse; LPN = licensed practical/vocational nurse; UNA = unlicensed nursing assistant.

### Process Measures

Process measures are often used by a facility to monitor its healthcare delivery system. It also has been observed by a number of researchers that improvement in process measures is not necessarily related to better outcomes (Ingraham et al., 2010; Jha, Joynt, Orav, & Epstein, 2012; Ryan, Nallamothu, & Dimick, 2012). For example, infection rates, which are outcome measurements, are dependent on multiple processes. A chain of successfully executed events must take place to reduce adverse patient outcomes. Looking at individual links in the chain may not predict the outcome since, like a chain, the patient's treatment is as successful as the weakest link. A facility's staff can be targeted to do the measured process as opposed to another and, thus, have no effect on the overall quality of the service provided. Outcomes that are dependent on a very few processes would be expected to be the most easily improved by implementing process measures. For example, the process measure of urinary catheter removal is an important measure since one of the most important risk factors in the development of urinary tract infections is the length of time the urinary catheter is left in place (Fakih et al., 2008).

### Outcome Measures

These measures have a comprehensive effect on patient care, and in order to have low rates of these adverse events, a facility must provide an appropriate level of nursing care. Outcome measures allow the facility to decide what protocols to follow and what level of staffing is necessary to achieve the optimal outcome. Many of these outcome measures have been implemented by CMS in current and future value-based purchasing initiatives (see **Table 1**). These initiatives include the nonpayment for care related to adverse events and the future rewarding and penalizing of a facility's reimbursement fee schedule based on performance on quality measurements. **Table 1** lists examples of nursing-sensitive outcome measures and estimates the costs of the adverse events they are designed to help prevent. All of these measures have been endorsed by the NQF, NDNQI, or CMS for value-based purchasing by CMS (2008).

## Nursing Workforce and Nursing-Sensitive Value-Based Purchasing

One of the major issues relating to the nursing workforce is not the number of nurses employed in the job market, but the number of hired and retained nurses. With the economic downturn in the United States, there are reports that nursing graduates are having difficulty finding employment (Terry & Whitman, 2011).

Research has shown that adequate nurse staffing levels lead to increased staff retention, job satisfaction, productivity, and better outcomes. However, new nurses report not being treated with respect as professionals, being required to work speeds similar to that of an experienced nurse with heavy workloads, and too many demands (Kovner & Brewer, 2011; Pellico, Brewer, & Kovner, 2009). Aiken et al. (2002) reported that increasing staffing levels from four patients per nurse to eight was associated with a 75% increase in the likelihood of job dissatisfaction and that 42% of nurses who were dissatisfied with their jobs intended to leave within 12 months, compared with 11% for nurses who had high job satisfaction. Furthermore, 73% of hospital nurses report inadequate staffing and over half of these nurses were considering leaving their job (ANA, 2010; Needleman et al., 2011). In an effort to understand hospital turnover rates, Kovner, Brewer, Fairchild, Poornima, Kim, and Djukic (2007) surveyed 1,195 newly licensed registered nurses (84% worked in hospitals) and found that 13% had changed their principal job after 1 year and 24% planned to leave their job in less than 2 years. One of the benefits of NSVBP may be an improvement in the practice environment leading to retention of nurses, which will help foster an experienced nursing workforce.

## Future Policy Evaluation and Research

Value-based purchasing is in its infancy. Devising an effective system that can transform our healthcare system can be viewed as a two-step process. First, truly meaningful measures must be identified. Second, these measures must be linked to effective financial incentives. Initiatives, which involve penalizing or rewarding overall payments to integrated healthcare delivery systems, are a focus of research in Accountable Care Organizations.

Process measures may not provide the increase in quality that was hoped for (Ingraham et al., 2010; Jha et al., 2012; Ryan et al., 2012). When they are implemented, a facility-wide analysis would be beneficial to make sure other areas of patient care are not sacrificed to augment the incentivized areas. Future research should focus on structure and outcome measures and their effect on increasing the quality of nursing care, along with the expected decrease in adverse patient outcomes and societal healthcare dollar savings.

Patient harm may also be caused during implementation of protocols in response to measures. For example, measurement of preoperative antibiotic administration may prompt a facility to streamline decision making and compliance by choosing to administer antibiotics to all surgical patients or to use the same antibiotic on all patients. Universal antibiotic administration risks long-term problems with increased bacterial resistance to achieve short-term gains. In the case of vancomycin, it has also been observed that preoperative use can increase infections in methicillin-resistant *Staphylococcus aureus*–negative patients (Gupta, Strymish, Abi-Haidar, Williams, & Itani, 2011). Thus, it is possible that a high score on a measure designed to reduce infections may actually be promoting them.

Societal costs include not only the healthcare costs to the payers and patients but also to society as a whole, including the patient's loss of employment, productivity, livelihood, and disability. In calculating cost effectiveness, many federal agencies estimate the value of a single life at $6.8 to $9.1 million (Appelbaum, 2011). Although healthcare data are available on savings to payers, few data are available on the financial impact on society as a whole. Further research could develop measures reflecting the true cost of debilitating patient outcomes.

Finally, measures can also spur research leading to the reduction in HACs. For example, ventilator-associated pneumonia and central line–associated bloodstream infections have a combined cost to the U.S. healthcare system of $1.7 to $4.18 billion (see **Table 1**). Studies on protocols in which nursing is a key component of the quality improvement team have shown that these infections can be reduced by 70% and approach zero in many institutions (Berenholtz et al., 2004, 2011). Nursing is an integral part of planning and implementation of protocols designed to lower the incidence of adverse events.

This timeline for implementation of NSVBP is short. In fiscal year 2013, CMS will implement hospital value-based purchasing using 12 process measures and patient experience of care survey measures (i.e., Hospital Consumer Assessment of Healthcare Providers and Systems). Measures are given different weights, and both achievement and improvement are utilized in scoring. Clinical process measures will be weighted 70% and patient experience of care weighted 30%. In fiscal year 2014, CMS will utilize three mortality outcome measures, eight HAC measures, and two AHRQ composite measures (CMS, 2011b). In future years, some measures will be dropped and others brought online. Thus, the question is not whether value-based purchasing should be implemented, but will nursing-sensitive measures be given proper weight and emphasis in the implementation of CMS's value-based purchasing initiative.

## Summary

As stated in the 2004 report from the Institute of Medicine (2004), “ … how well we are cared for by nurses affects our health, and sometimes can be a matter of life or death” (p. 2). To maintain and achieve quality health care, a hospital must have quality nursing. A short-term fix for facility budgetary shortfalls is to decrease nurse staffing costs. Reductions in nurse staffing reduces the quality of healthcare delivery and increases adverse events.

The intent of NSVBP is to financially incentivize a change in conditions wherever the economic benefit of increasing nursing staff to the purchaser or patient is greater than that to the hospital or provider. This would rebalance the financial equation in favor of improved patient care by financially rewarding hospitals that increase nursing staff and would lower societal healthcare costs by producing better patient outcomes. NSVBP has the potential to help reverse hospital understaffing, to improve the quality of delivered health care, and to lower societal healthcare costs.

## Clinical Resources

1. American Nurses Foundation. Medicare/Medicaid: Recognizing RNs' contributions: http://www.rnaction.org/site/PageServer?pagename=CUP_Arch_080408_ag1_medicomments&ct=1
2. Center for Medicaid & Medicare. The official website for the Medicare hospital value-based purchasing program: https://www.cms.gov/Hospital-Value-Based-Purchasing/
3. Disparities in healthcare utilization among Medicare referral regions: http://www.dartmouthatlas.org/
4. Healthcare associated infections, adverse outcomes, and healthcare reform: http://www.healthwatchusa.org
5. Listing of organizations designed to promote healthcare quality: http://www.nursingworld.org/MainMenuCategories/ThePracticeofProfessionalNursing/PatientSafetyQuality/Quality-Organizations
6. National Quality Forum. Definitions and metrics of quality measures: http://www.qualityforum.org
7. Agency for Healthcare Research and Quality. Theory and reality of value-based purchasing and measuring healthcare quality: http://www.ahrq.gov/qual/meyerrpt.htm and http://www.ahrq.gov/qual/measurix.htm
